# Cockroaches as Reservoirs, Vectors, and Potential Sentinels of Multidrug-Resistant Bacteria in Ugandan Communities: A Retrospective Analysis

**DOI:** 10.1155/ghe3/5940509

**Published:** 2025-01-19

**Authors:** Steven Kakooza, Paul Ssajjakambwe, Rebecca Nalubega, Betty Namazi, Aisha Nantume, Geoffrey Ssentamu, Esther Nabatta, David Nalumenya, Mariam Wanyana, Damien F. N. Munyiirwa, Dorcus Namuyinda, Sayaka Tsuchida, Kazunari Ushida, John Baligwamunsi Kaneene

**Affiliations:** ^1^Central Diagnostic Laboratory, College of Veterinary Medicine, Animal Resources and Biosecurity, Makerere University, Livingstone Road, Kampala, Uganda; ^2^Department of Veterinary Pharmacy, Clinics and Comparative Medicine, College of Veterinary Medicine, Animal Resources and Biosecurity, Makerere University, Kampala, Uganda; ^3^National Agricultural Research Organization, 14 Kitaasa Road, Entebbe, Uganda; ^4^Department of Biomolecular and Biolaboratory Sciences, College of Veterinary Medicine, Animal Resources and Biosecurity, Makerere University, Veterinary Animal House, Kampala, Uganda; ^5^Centre for Biosecurity and Global Health, Makerere University, Kampala, Uganda; ^6^Department of Environmental Biology, College of Bioscience and Biotechnology, Chubu University, Kasugai, Aichi, Japan; ^7^Center for Comparative Epidemiology, College of Veterinary Medicine, Michigan State University, 736 Wilson Road, Room A109, East Lansing, Michigan 48824, USA

**Keywords:** antimicrobial resistance, bacteria, cockroaches, pathogen reservoirs and vectors, public health, sentinels, Uganda

## Abstract

**Background:** Cockroaches could play a role in the transmission dynamics of antimicrobial-resistant bacteria (ARB) at variable interfaces in Ugandan communities, acting as both reservoirs and vectors. This study investigated the burden and diversity of ARB carried by cockroaches in human settlements in Uganda, so as to understand their role in the spread of these pathogens and their potential as sentinels in antimicrobial resistance (AMR) surveillance programs.

**Materials and Methods:** A retrospective analysis was conducted on two unpublished studies by Makerere University students. Study one and study two sampled 58 and 110 cockroaches, respectively, from secondary schools in Kampala. Cockroach species identification was determined based on physical characteristics. Bacterial isolation and characterization were performed through microbiological analyses including standard culture methods, biochemical tests, matrix-assisted laser desorption/ionization time-of-flight mass spectrometry (MALDI-TOF MS), disc diffusion method, and polymerase chain reaction (PCR).

**Results:** Majority of the cockroaches (over 80%) were *Periplaneta americana.* Multidrug resistance (MDR) was prevalent among the isolates, with over 30% of the isolates being resistant to three or more antibiotic classes. Specifically, MDR (over 90%) was rampant in the extended spectrum beta-lactamase (ESBL)– or AmpC-producing *Escherichia coli* and enterococci isolates. Critical World Health Organization (WHO) priority pathogens, such as ESBL-/AmpC-producing Enterobacteriaceae, and carbapenem-resistant *E. coli*, were also identified. The most abundant resistance determinants (tetracycline and sulphonamide) were *tetA, sul1,* and *sul2* for *E. coli*, and *tetM* and *tetL* for enterococci.

**Conclusion:** The findings accentuate the potential role of cockroaches: (1) in transmitting multidrug-resistant bacteria at the human–animal–environment interface and (2) as sentinels in the surveillance of community-generated AMR.

## 1. Introduction

The emergence and spread of antimicrobial resistance (AMR) in bacteria is a significant threat to global public health, compromising the effectiveness of antibiotics in treating common infections [1, 2]. In Uganda, human health studies [3–5] have highlighted the emergence of AMR (in bacteria like *Escherichia coli* and *Klebsiella pneumoniae*) linked to commonly used World Health Organization (WHO)–classified critical or highly important antibiotics, such as third-generation cephalosporins, carbapenems, and fluoroquinolones. The estimated burden of such clinically significant multiple antibiotic-resistant Gram negatives could exceed 20.0%, depending on the geographical region. Similarly, animal health studies [6–9] have revealed that food animals are significant hosts of multidrug-resistant commensal and clinical *E. coli*, with prevalence estimates ranging from 17.5% to over 50%. There remains a glaring gap in data from the environmental health side; however, a recent study [10] reported on colonization of water sources by multidrug-resistant organisms (over 50%). These trends across multiple One Health components emphasize the urgent need for effective surveillance programs to track the spread of AMR and identify potential reservoirs of resistant bacteria, thus playing a vital role in preventing future pandemics. The global impact of the recent COVID-19 pandemic highlighted an urgent imperative to reduce the risk of emergent zoonotic pandemics or epidemics. This necessitates fundamental changes in our interaction with nature [11], including minimizing human contact with potential disease vectors and removing possible transmission points with high spillover potential.

In the context of One Health, biological species that live near humans and animals ought to be closely monitored, especially if they could pose health risks such as AMR [12]. Pests like house flies, cockroaches, and many others have been implicated as vectors of infectious threats in humans [13–15]. Cockroaches (Order: Blattodea) are ubiquitous insects globally, with over 4,000 species [16, 17]. Among the most prevalent species are the American cockroach (*Periplaneta americana*) and German cockroach (*Blattella germanica*) [17, 18], along with others such as *Blatta orientalis* and *Supella longipalpa*.

Cockroaches share common characteristics such as a flattened oval body, biting and chewing mouth parts, elongated antennae, and legs. They have species-specific reproductive behaviors, typically laying 30–50 eggs per ootheca. The American cockroach produces 10–20 oothecae with 14–16 eggs each, while the German cockroach produces 4–8 oothecae with 30–40 eggs each. They are most active and reproduce more during warmer months, peaking from late spring to summer in temperate climates, and reproducing year-round in tropical and subtropical regions [19, 20]. As scavengers, cockroaches feed on a wide range of organic matter, making them intolerable pests infesting human settlements, including homes, food-handling establishments, hospitals, and other public facilities [21].

The habitats of cockroaches, combined with their body structure and omnivorous feeding, do not only favor sustenance of their numbers but also make them prone to harboring pathogens rendering them potential disease reservoirs and vectors [22]. Pathogen transmission mechanisms include microbes on their outer cuticle and excreta dropped on human-consumed food and other surfaces. Due to their ecological niche, cockroaches could be promising candidates for AMR surveillance even in Uganda.

Various studies have isolated bacterial species of public health significance from the external surface and digestive tract of cockroaches, including staphylococci, streptococci, enterococci*, Escherichia coli, Klebsiella* spp., and *Pseudomonas aeruginosa* [22–24]. These studies documented the carriage of AMR bacteria by cockroaches, ultimately highlighting their potential role as vectors. Many of these bacteria detected are classified under the critical, high, or medium priority categories in the WHO global priority pathogens list (PPL). In another study, further characterization of the cockroach bacterial isolates also revealed noxious cliques of antimicrobial resistant bugs, such as extended spectrum beta-lactamase (ESBL)–producing bacteria [24].

Cockroaches, occupying a synanthropic niche (live in close association with people and benefit from their surroundings and activities) in Ugandan communities, can potentially facilitate the transmission of antimicrobial-resistant bacteria (ARB) through various interfaces, including human–cockroach, cockroach–environment, and cockroach–animal interactions. Despite evidence from other regions, the role of cockroaches in the transmission dynamics of ARB in the context of Uganda remains largely unexplored. This study aimed to address this gap in knowledge by investigating the burden and diversity of ARB carried by cockroaches in secondary schools in Kampala, Uganda. We hypothesize that cockroaches in these settings are carriers of diverse groups of multidrug-resistant bacteria and that they could serve as reservoirs and sentinel vectors for monitoring the spread of these pathogens. Understanding the potential of cockroaches as reservoirs and sentinel vectors of resistant bacteria is crucial for developing effective AMR surveillance programs and mitigating the public health risks associated with these common pests.

## 2. Methods and Materials

### 2.1. Study Design, Setting, and Samples

This retrospective study analyzed findings from two unpublished gray literature studies by Bachelor of Biomedical Laboratory Technology students at Makerere University, Uganda. These studies investigated cockroaches as vectors of public health pathogens in Kampala, Uganda (latitude: 0.347596, longitude: 32.582520), which has five divisions: Central, Kawempe, Makindye, Nakawa, and Rubaga.

Study one, a cross-sectional study conducted between December 2019 and April 2020, sampled 58 cockroaches from two secondary schools in Central and Nakawa divisions. Study two, conducted between July and September 2022, sampled 110 cockroaches from two secondary schools in urban and periurban Kampala. Both studies purposively selected schools and sample sizes based on accessibility and feasibility of cockroach collection.

### 2.2. Sample Collection and Analyses

#### 2.2.1. Sample Collection

The two studies used modified methods from a past entomological study [25]. Live cockroaches were collected from vermin glue traps using sterile forceps. These traps were placed in toilets, kitchens, and dormitories at 6:00 pm and collected by 7:00 am the next morning, following a previous study [26]. Samples were transported in 50 mL sterile air-tight falcon tubes on ice to the Central Diagnostic Laboratory (CDL), College of Veterinary Medicine, Animal Resources and Biosecurity (CoVAB), Makerere University.

#### 2.2.2. Cockroach Species Identification and Sexing

Cockroaches were identified based on physical characteristics [27]. *Blattella germanica* (German cockroaches) were 1.1–1.6 cm long and light brown with two dark stripes behind the head. *Periplaneta americana* (American cockroaches) were 3.0–4.0 cm long and reddish-brown, with wings for gliding [28]. Sex differentiation used body shape (males: smaller), abdomen shape (females: boat-shaped and blunt last segment), wings (males: larger and extended beyond the abdomen), and antennae size (females: larger).

#### 2.2.3. Sample Processing

Cuticle sample processing was done by washing the surface of the cockroach and dissection methods to be able to detect both the internal and external microbial carriage. 20 mL of sterile 0.85% physiological saline was added to the tubes with the cockroaches and shaken gently for 2 minutes.

##### 2.2.3.1. Dissection Technique for Gut Extraction

After external body washing, the external surfaces of cockroaches were decontaminated with 70.0% ethanol and rinsed with sterile saline to remove ethanol traces. The alimentary tracts were then aseptically dissected [29]. The process involved clipping wings, fixing specimens dorsally on a dissecting tray, and exposing the thoracic and abdominal cavities. Sterile distilled water was used to remove fat bodies and tracheae, exposing the internal organs, which were then picked and macerated with saline solution in a sterile 2 mL Eppendorf tube [30].

##### 2.2.3.2. Isolation of Bacteria of Public Health Interest

The cultures focused on isolation and characterization of some pathogens appearing on the WHO's global PPL [31], including *Escherichia coli* (carbapenem-resistant and ESBL-producing *E. coli)* (critical priority) and *Staphylococcus aureus* (methicillin-resistant *S*. *aureus*) (high priority).

*E. coli*: 1 mL of both the saline wash and the organ homogenate was added to separate tubes with 9 mL of Buffered Peptone Water (BPW) (Conda Laboratories, Spain) and incubated for 24 h at 37°C aerobically. After incubation, cultures were streaked onto MacConkey agar (Conda Laboratories, Spain). Single intense pink colonies were subcultured on nutrient agar (Merck, England) and incubated at 37°C for 18 h [6, 7]. Colonies were further identified by matrix-assisted laser desorption/ionization time-of-flight mass spectrometry (MALDI-TOF MS)–based systems (Section 2.3.2).

*S. aureus:* Following protocols [32, 33], 1 mL of the saline wash and organ homogenate were enriched in 9 mL of 7.0% NaCl nutrient broth and incubated for 24 h at 37°C aerobically. Enriched cultures were streaked onto 5.0% defibrinated sheep blood agar (Conda Laboratories, Spain). Medium-sized white, gray, or yellow double hemolytic colonies were subcultured on nutrient agar and incubated at 37°C for 18 h. Colonies were identified as *S. aureus* by catalase (positive), nitrate (positive), urease (positive), mannitol (positive), maltose (positive), and coagulase (positive) tests.

Enterococci: Using modified [34] methods, 1 mL of both the saline wash and organ homogenate was added to separate tubes with 10 mL BPW and incubated for 24 h at 37°C aerobically. Enrichment cultures were streaked onto Enterococcosel Agar (Becton Dickinson, USA). Presumptive *Enterococcus* spp. colonies with brown-black halos were subcultured on 5.0% sheep blood agar and subjected to Gram staining (Gram-positive cocci), catalase (negative), and MALDI-TOF MS typing (Section 2.3.2). Proteomic typing was done for 28 archived enterococci from study one.

##### 2.2.3.3. Antimicrobial Susceptibility Testing

Antimicrobial susceptibility tests were conducted on *E. coli* and enterococci using the disc diffusion method (Kirby–Bauer) according to the CLSI guidelines [35]. Pure colonies were resuspended in 0.85% saline and calibrated to a 0.5 McFarland standard. Mueller–Hinton agar (MHA) plates were inoculated with the suspension and antibiotic discs applied. For enterococci, MHA was spiked with 5.0% defibrinated sheep blood [36]. Plates were incubated at 37°C for 18–24 h, and zones of inhibition were measured and compared to standard charts to determine resistance or susceptibility. Antimicrobials used included ampicillin (10 µg), ceftriaxone (30 µg), chloramphenicol (30 µg), ciprofloxacin (5 µg), gentamicin (10 µg and 120 μg), tetracycline (30 µg), trimethoprim sulfamethoxazole (25 µg), linezolid (30 µg), erythromycin (15 μg), meropenem (10 µg), and imipenem (10 µg). Multidrug resistance (MDR) was defined as resistance to at least one agent in three or more antimicrobial families [37]. Quality control used standard strains of *E. coli* ATCC 25922 and *S. aureus* ATCC 25923.

### 2.3. Complementary Microbial Analyses

The two studies focused on culture and antimicrobial sensitivity testing of selected bacteria. From the archived specimens, bacteria were recovered and characterized using automated microbiology and polymerase chain reaction (PCR) as explained below.

#### 2.3.1. Isolation of ESBL- or AmpC-Producing Gram-Negative Bacteria

Detection of ESBL- or AmpC-producing bacteria was done using 124 archived samples (guts and cuticles: 82; generic *E. coli*: 42) from study one. Modified methods from a previous study (6) were used. Samples were pre-enriched in BPW (Conda Laboratories, Spain) and incubated at 37°C for 24 h. Cultures were streaked on MacConkey agar (Conda Laboratories, Spain) with 1 mg/L of cefotaxime and incubated at 37°C for 24 h. All colonies (nonlactose fermenting and lactose fermenting) were subcultured and identified by MALDI-TOF MS.

#### 2.3.2. Identification of Isolates by MALDI-TOF MS

Species-level identification was performed using MALDI-TOF MS, MicroFlexTM, Bruker Daltonics, United States [38, 39]. This was applied for *E. coli*, enterococci, and ESBL-/AmpC-producing Gram-negative isolates from study one. The sensitivity and specificity for identifying *Enterococcus* strains using MALDI-TOF MS ranged from 80.0% to 96.7% and 90.0% to 98.1%, respectively [40]. Detection of Enterobacteriaceae producing ESBLs was reported at 99.0% sensitivity [41]. In brief, bacterial colonies were spread on a MALDI target plate, air-dried, overlaid with Bruker IVD Matrix HCCA, and air-dried for 5 minutes. The target plate was then imported into the Bruker MicroFlex instrument operated using FlexControl 3.0 software. Instrument calibration was performed using Bruker's Bacterial Test Standard (BTS, Bruker Daltonics).

#### 2.3.3. Molecular Detection of AMR Markers

The molecular basis of tetracycline and sulfonamide resistance in *E. coli* and enterococci isolates was studied using colony PCR [42] with gene-specific primers and conditions from previous studies [43, 44]. Both naïve and strains resistant to tetracycline and sulfonamides were tested. Steps involved recovery in nutrient broth, PCR, agarose gel electrophoresis, and PCR product illumination. Positive control DNA with targeted genes was used during PCR and visualization in 2% agarose gels.

### 2.4. Data Analysis

The studies reported that data were entered in Microsoft Excel and analyzed using SPSS version 25.0. Descriptive statistics summarized the data using percentages. The frequency of selected bacteria species by address, site sampled, sex, alimentary tract, and external surface was computed as the proportion of positive cockroaches from the total sampled. The frequency of MDR was calculated as the proportion of isolates resistant to at least three antimicrobial classes. The multiple antibiotic resistance index (MARI) for each isolate was calculated using the formula a/b, where a is the number of antibiotics to which the isolate was resistant, and b is the total number of antibiotics tested [45]. The mean MARI of each bacterial group was computed as the sum of MARI values divided by the total isolates.

### 2.5. Ethical Considerations

Approval and permission for the studies were obtained from the Institutional Review Board of the School of Biosecurity, Biotechnology and Laboratory Sciences, CoVAB, Makerere University. Permission to conduct research on the archived biologics was received from the management of the Central Diagnostic Laboratory, CoVAB, Makerere University, where the testing for both studies had been done.

## 3. Results

### 3.1. Characteristics of Cockroaches

Study one: A total of 58 cockroaches were collected from two schools (mixed sex school; 30, single sex girls' school; 28) located in Kampala. The specimens were sampled from three habitats: the dormitory (*n* = 17), kitchen (*n* = 20), and toilet (*n* = 21), of which 81.0% (*n* = 47) were *P. americana* and 19.0% (*n* = 11) were *B. germanica.* 43.1% (25/58) were characterized as male and 56.9% (33/58) as female.

Study two: 110 cockroaches were collected from two schools located in and periurban Kampala. From the collection, 100/110 (90.9%) were *P. americana* and 10/110 (9.1%) were *B. germanica*. These specimens were collected from the dining hall, dormitories, and toilets.

### 3.2. Bacterial Carriage in Cockroaches

Study one: From the sampled cockroaches (*n* = 58), specimens from the same habitat were pooled for analyses of cuticle surface bacteria. Overall, a total of 82 samples were analyzed: cuticle washes of pooled specimens (*n* = 24) and the guts (*n* = 58). *Escherichia coli*, *Staphylococcus aureus*, and *Enterococcus* spp. were tested across both sample types. *E. coli* was detected in nearly half of the gut samples (48.3%) and over half of the cuticle washes (58.3%), while *S. aureus* was absent in all samples (0.0%). In contrast, *Enterococcus* spp. was consistently present in all cockroaches sampled (100%), regardless of sample type or species. Interestingly, *B. germanica* had a higher *E. coli* carriage (72.7%) compared to *P. americana* (42.6%), and no gender differences were observed for *Enterococcus* spp., though males had a slightly higher prevalence of *E. coli* than females (51.5% vs. 44.0%).

Study two: From the 110 specimens collected, only 66 gut samples were extracted and 15 pooled cuticle samples making a total of 81 samples. *Enterococcus* spp. and *E. coli* were isolated from the cockroach samples, with a high recovery of *E. coli* from both the cuticle (93.3%) and gut (78.8%), while *Enterococcus* spp. was less frequently detected (cuticle: 6.6%, gut: 21.2%). A detailed breakdown of bacterial species recovery in both studies is provided in Table 1.

### 3.3. MALDI-TOF MS Analyses of Bacteria Subset of Samples

This was only done with isolates associated with study one. All the presumptive *E. coli* isolates from study one (*n* = 42) that had been subcultured on MacConkey agar (without antibiotics) and biochemically identified were confirmed to be *E. coli* using MALDI-TOF MS methods.

Only 28 presumptive enterococci strains were speciated giving the following: *Enterococcus faecalis, Enterococcus faecium, Enterococcus casseliflavus, Enterococcus hirae, Enterococcus thailandicus,* and *Enterococcus villorum*. Most of these strains were *E. faecalis* (14; 50.0%) and *E. faecium* (6, 21.4%) (Table 2).

ESBL-/AmpC-producing Gram negatives: Of the 83 study one specimens, 43 (51.8%) screened positive for various species of ESBL-/AmpC-producing bacteria. Forty-three strains were further identified giving the species; *E. coli*, *Klebsiella pneumoniae*, *Klebsiella variicola*, *Citrobacter freundii*, *Aeromonas caviae,* and *Morganella morganii*. Many of these strains were *E. coli* (25; 58.1%) and *K. pneumoniae* (12; 27.9%) (Table 3).

### 3.4. AMR in Generic *E. coli,* Enterococci, and ESBL-/AmpC-Producing *E. coli* Isolates

Study one: Of the 42 generic *E. coli* isolates, most isolates were resistant to ampicillin (69.0%), followed by tetracycline (47.6%), and trimethoprim sulfamethoxazole (38.1%). Minimal resistance was recorded with gentamicin (0.0%), imipenem (0.0%), and ciprofloxacin (7.1%). In this population, 15 (35.7%) isolates were screened to be ESBL-/AmpC-producing using MacConkey with cefotaxime.

Altogether, 40 ESBL-/AmpC-producing *E. coli* (25 from the guts and cuticle samples, and 15 from the 42 generic *E. coli*) were tested. The majority were resistant to ampicillin (80.0%), followed by ceftriaxone (77.0%) and trimethoprim sulfamethoxazole (72.5%). Minimal resistance was seen with imipenem (7.5%), followed by chloramphenicol (30.0%) and ciprofloxacin (35.0%). Among 42 enterococci isolates (randomly selected from 82 strains), the majority were resistant to linezolid (92.9%) and tetracycline (88.1%). Minimal resistance was majorly recorded with gentamicin (9.5%). The latter *Enterococcus* spp. isolates were detected as high-level gentamicin-resistant (HLGR).

The prevalence of MDR in generic *E. coli,* enterococci, and ESBL-/AmpC-producing *E. coli* isolates was 38.1%, 100%, and 97.6% (Table 3). The following MDR classes were found in *E. coli*: MDR3 (12), MDR4 (2), MDR5 (1), and MDR6 (1). For enterococci, MDR was characterized into these classes: MDR3 (2), MDR4 (10), MDR5: (15), MDR6: (11), and MDR 7 (3), whereas for ESBL-/AmpC-producing *E. coli*, these MDR classes were profiled as MDR3 (10), MDR4 (8), MDR5 (9), MDR6 (8), and MDR7 (2). Antibiotic resistance profiles of *E. coli* and enterococci isolates of study one are summarized in Table 4.

Study two: As shown in Table 5, antimicrobial sensitivity testing was done for 66 *Enterococcus* spp. and 15 *E. coli* isolates against 6 antimicrobials of clinical significance. Enterococci showed the highest phenotypic resistance for trimethoprim sulfamethoxazole, tetracycline, gentamicin, and ciprofloxacin as 66/66 (100%), 64/66 (96.97%), 66/66 (100%), and 61/66 (92.42%), respectively. *E. coli* showed the highest phenotypic resistance to trimethoprim sulfamethoxazole, tetracyclines, and ampicillin as 13/15 (86.67%), 10/15 (66.67%), and 13/15 (86.67%), respectively.

### 3.5. Bacterial Genetics of AMR

Study one: The analysis of resistance genes in *E. coli* and enterococci revealed notable differences in the prevalence of tetracycline and sulfonamide resistance genes. Among commensal *E. coli*, the *TetA* gene was detected in nearly half of the samples (46.7%), while the other tetracycline resistance genes *Tet* (*B, C, D, E, G*) were absent (0.0%). Sulfonamide resistance was also observed, with *sul1* and *sul2* genes having similar prevalence (26.7%), while *sul3* was the least detected (20.0%). The ESBL-/AmpC-*E. coli* collection showed higher prevalence for both tetracycline and sulfonamide resistance genes compared to commensal *E. coli*. The *TetA, TetB*, *sul1, sul*2, and *sul3* genes were detected in 60.0%, 32.0%, 52.0%, 32.0%, and 24.0% of the samples, respectively, whereas the *Tet* (*C, D, E, G*) genes were not detected (0.0%). In enterococci, *TetM* and *TetL* genes were highly prevalent across the samples (82.5% and 85.0%, respectively).

Study two: The detection of *TetA* and *TetB* in *E. coli* was found at 13.3% and 6.7%, whereas the *Tet* (*C, D, E, G*) genes were not detected (0.0%). The prevalence of *sul1* and *sul2* was both at 26.7% and the *sul3* gene was not detected at all (0.0%). Additionally, the *TetM* gene was present at a prevalence of 19.7% in enterococci, while *TetL* was absent (0.0%). A detailed comparison of the prevalence of resistance genes across both studies can be found in Table 6.

## 4. Discussion

In this study, two cockroach species, including *P. americana* and *B. germanica*, were identified. *Periplaneta americana* was the most frequent cockroach (over 80%), followed by *B. germanica* (less than 45%), a finding consistent with previous studies [12, 46, 47].

The bacterial organisms carried by the cockroaches in this study have also been reported by studies done in France, India, Iran, Bangladesh, Ethiopia, and Ghana [21, 29, 46, 48–52]. This analysis reports variable prevalence rates of *Enterococcus* spp. in cockroaches (100% in study 1, and 6.6% and 21.2% in study 2). Compared to research done in Ghana [49], which reported a prevalence of less than 4%, this study reported higher values. In our analysis, both studies detected *E. coli*, although at varying frequencies (48.3% and 58.3% in study 1, and 93.3% and 78.8% in study 2). Earlier studies in Bangladesh [29], Iran [52], and Ethiopia [53] detected *E. coli* but at lower rates compared to our study. Study one did not isolate *S. aureus*, unlike previous studies [21, 54–56] where cockroaches from various environments often had *S. aureus*, a common nosocomial bacterium. Enterococci were recovered more frequently than *E. coli*, possibly due to different selective media; MacConkey agar allows growth of various Gram-negative bacteria, while Enterococcosel Agar is more selective for enterococci. Previous research [21] reported lower recovery of enterococci, likely influenced by detection methods, as both enterococci and *E. coli* are common commensals in various hosts and environments [57, 58]. The prevalence variations could be due to factors like differences in geographical locations, laboratory methods for bacterial detection, and cockroach species that were studied. Additionally, while this study focused on secondary schools, other research has sampled cockroaches from hospitals, restaurants, and households, potentially leading to variations in bacterial profiles due to different environmental pressures.

The WHO has articulated a PPL to provide strategic direction to research and develop new antimicrobials against resistant bacteria [59]. The list included 12 families of bacteria that were implicated to pose the greatest threat to human health and further grouped into three priority groups (critical, high, and medium). In this study, *E. coli*, enterococci, and ESBL-/AmpC-producing Gram-negative bacteria were detected. Further screening of commensal *E. coli* and ESBL-/AmpC-*E. coli* by phenotypic AMR diagnostics revealed low-moderately high prevalence of carbapenem-resistant *E. coli* (critical priority) (study one: 7.5%, study two: 20.0%) and relatively high numbers of ESBL-/AmpC-producing *E. coli* (critical priority) (study one: 30.5% and 35.7%) and no methicillin-resistant *S. aureus* (high priority) across the studies. In Uganda, recent AMR studies [3, 6, 7, 60, 61] have mainly focused on the epidemiology in humans, animals, food, and bacteria, but with less surveillance on new reservoirs and vectors of ARB being created in the community due to anthropogenic practices. Elsewhere, studies [62] have shown that antimicrobial residues, antibiotic resistance genes (ARGs), and ARB are continuously introduced from hospital wastewater and municipal waste into the environment, making it a reservoir of infectious threats. Waste microbiology could reflect community and hospital-generated AMR, ultimately supporting sentinel AMR surveillance as seen in a study [63] that found ESBL *E. coli* in hospital sewage. Some individuals, particularly those with omnivorous diets like cockroaches, can access contaminated waste sites, potentially becoming reservoirs of pathogens. The identification of critical WHO PPL pathogens carried by cockroaches emphasizes their role as potential reservoirs and sentinelsfor human exposure to these antimicrobial resistant bacteria [64, 65]. Previous studies [66, 67] also found critical WHO bacteria like ESBL Enterobacteriaceae carried by cockroaches. However, to better understand the transmission dynamics of resistant bacteria at variable human–animal–cockroach–environment interfaces, research should employ advanced genomic technologies, such as sequencing and bioinformatics, to study bacterial strains from humans, cockroaches, animals, and the environment within synanthropic niches.

Resistance of commensal *E. coli* from cockroaches analyzed in this study was mainly seen with ampicillin, tetracycline, and trimethoprim sulfamethoxazole, a finding consistent with previous studies [21, 68]. Resistance in enterococci was predominant with linezolid and tetracycline, a finding in disagreement with previous results by Bouamamaa et al. [55]. These disparities are likely to be linked with the differences in AMR detection methods and sample size variations.

Enterococci exhibiting HLGR is a threat as these multidrug-resistant strains have been widely documented as a cause of nosocomial infections [69]. Gentamicin in combination with a cell wall active agent is regularly used against serious enterococcal infections [70]. However, the emergence of high-level aminoglycoside resistance (HLAR) is responsible for loss of synergy between agents active on the cell wall and aminoglycosides. This is the first study to report a prevalence of HLGR enterococci of 9.5% in cockroaches.

Among the ESBL-/AmpC-producing *E. coli*, high resistance rates (over 60%) were found for tetracycline, ampicillin, trimethoprim-sulfamethoxazole, gentamicin, and ceftriaxone. There were no previous studies on cockroach AMR to compare with these findings. Imipenem showed the lowest resistance rate (7.5%) in this study. Thus, carbapenems can be used as an option in cases of community-onset of related infections as previously recommended [71]. However, three out of 40 isolates were carbapenem-resistant, posing a public health threat if these strains cause human infections. For minor ESBL infections, noncarbapenem antibiotics, such as beta-lactamase inhibitors (e.g., clavulanic acid, sulbactam, tazobactam, avibactam, and vaborbactam), can be considered, often in combination with other classes.

Among all bacterial groups, over 30% of the isolates were MDR strains, resistant to 3–7 antibiotics, consistent with previous studies [21, 29]. However, over 90% of the ESBL-/AmpC-producing *E. coli* and enterococci isolates were MDR, which is concerning due to their association with nosocomial and community-acquired infections [72, 73]. Study one found low resistance rates (0%–7%) to gentamicin, imipenem, chloramphenicol, and ciprofloxacin in commensal *E. coli*, and only imipenem for ESBL-/AmpC-producing *E. coli*. This highlights the need for more AMR surveillance in different pathogen groups and specific AMR phenotypes, using risk-based evidence for targeted drug discovery. Bacteria with a MARI of 0.2 or higher (as reported by this study) come from high-risk contamination sources with frequent antibiotic use [74, 75], explaining the high MDR in our isolates. Cockroaches feeding on contaminated waste may be exposed to antimicrobials, exerting positive selection pressure on their gut microbiota, leading to increased AMR gene carriage. Previous studies identified the housefly gut as an environment for transferring AMR genes among bacteria [13, 76], which could similarly occur in cockroaches.

The ARGs of bacteria can be found in diverse environments such as the gut, soil, air, and water [77, 78]. In this study, we report on the detection of ARGs in *E. coli* and enterococci isolated from cockroach guts and cuticles. The most prevalent tetracycline and sulfonamide resistance determinants were *tetA, sul1,* and *sul2* for *E. coli*, and *tetM* and *tetL* for enterococci. We could not find other studies that detected similar ARGs from cockroach bacteria to compare with these findings.

Characterization of ESBL-/AmpC-producing isolates using MALDI-TOF MS revealed this phenotype in various Gram-negative bacteria genera, such as *Morganella*, *Aeromonas*, and *Citrobacter*, though *E. coli* and *K. pneumoniae* were most common [6, 66, 79], consistent with our findings.

The MALDI-TOF MS identified a portion of the microbiota, but future studies could benefit from metagenomics and next-generation sequencing to better understand the gut microbiome and resistome of these pests. While insects like flies and cockroaches are known mechanical vectors, emerging evidence suggests they may also contribute to the horizontal and vertical transmission of bacterial ARGs, potentially fostering disease reservoirs and vectors. This aspect was not explored in our study due to resource constraints. Our sampling was predominantly from school environments, which may not fully represent other critical settings such as hospitals or restaurants or households. This could limit the generalizability of our findings. The sample sizes used were also not robust enough to improve the statistical vigor of our results. Additionally, our focus on specific pathogens, while valuable, may have led us to overlook other significant bacteria that could also be implicated as multidrug resistant. To better understand the transmission pathways of resistant bacteria, future studies should investigate the genetic relatedness of cockroach strains to clinical and nonclinical bacteria isolated from humans or animals living in cockroach-infested areas, as well as the shared environmental niche. This will allow for a more comprehensive investigation of the spread of resistant bacteria and help determine whether cockroaches act as a bridge between these different One Health components. Despite these limitations, our findings highlight the potential role of cockroaches in the transmission dynamics of ARB, warranting further research using more diverse sampling sites, robust sample sizes, and advanced genomic techniques to better understand their impact on public health.

The findings highlight significant public health implications of cockroaches as reservoirs and potential vectors of multidrug-resistant bacteria, creating a demand for a comprehensive approach to effectively manage and eliminate cockroach populations in schools. This includes improving sanitation and hygiene practices to cover regular cleaning and proper food waste disposal [21, 80]; exclusion by sealing entry points like cracks and crevices in walls, floors, and ceilings; using chemical and biological control measures; educating staff and students on actions to prevent cockroach infestations; and conducting regular monitoring and inspections to track populations [81]. These combined efforts will significantly reduce cockroach infestations, ensuring a healthier and safer school environment. The study suggests that cockroaches can serve as sentinel insect species for monitoring the spread of AMR in the community. This could inform public health surveillance programs and help in the early detection of resistant bacterial strains. Further studies should validate this role, using a One Health approach to quantify ARB and ARGs, and compare strains in humans, animals, and the environment.

## 5. Conclusion

This study confirmed that cockroaches can be a potential reservoir and vector of resistant pathogens and ultimately could play a role in the mechanical dispersal or through fecal dispersal of such bacteria or ARGs between the environment and human beings. With this information, the insect can be studied more to be validated as a biological species to monitor community-generated AMR. The bacteria isolated have been implicated in the spectrum of common community infections, including nosocomial infections such as urinary tract infections, respiratory pneumonia, surgical site wound infections, bacteremia, gastrointestinal, and skin infections. The high prevalence rates of resistant organisms predict a potential economic threat of treatment failures when managing community infections caused by cockroach-mediated microbes. Consequently, community health centers need to embrace good infectious disease diagnostic stewardship where perilous antibiotic stewardship practices, such as blind treatment, can be replaced by adopting accurate diagnostics such as bacterial culture and sensitivity tests for better prognostic outcomes. Also, proper prevention and control measures against cockroaches that can vector pathogens must be practiced in human dwellings to limit associated infections.

## Figures and Tables

**Table 1:** Bacterial species isolated from cockroach samples.

Study	Sample type	Number of samples	Bacteria	Frequency (%)
Study one	Guts	58	*E. coli*	28 (48.3)
			*S. aureus*	0 (0.0)
			*Enterococcus* spp.	58 (100.0)
	Cuticle wash	24	*E. coli*	14 (58.3)
			*S. aureus*	0 (0.0)
			*Enterococcus* spp.	24 (100.0)
	*P. americana* guts	47	*E. coli*	20 (42.6)
			*S. aureus*	0 (0.0)
			*Enterococcus* spp.	47 (100.0)
	*B. germanica* guts	11	*E. coli*	8 (72.7)
			*S. aureus*	0 (0.0)
			*Enterococcus* spp.	11 (100.0)
	Male cockroaches	33	*E. coli*	17 (51.5)
			*S. aureus*	0 (0.0)
			*Enterococcus* spp.	33 (100.0)
	Female cockroaches	25	*E. coli*	11 (44.4)
			*S. aureus*	0 (0.0)
			*Enterococcus* spp.	25 (100.0)

Study two	Guts	66	*E. coli*	52 (78.8)
			*Enterococcus* spp.	14 (21.2)
	Cuticle wash	15	*E. coli*	14 (93.3)
			*Enterococcus* spp.	1 (6.6)

**Table 2:** Proportions of *Enterococcus* species identified by MALDI-TOF MS (*n* = 28).

Enterococci	Frequency	Percentage (%)
*Enterococcus faecalis*	14	50.0
*Enterococcus hirae*	4	14.3
*Enterococcus faecium*	6	21.4
*Enterococcus casseliflavus*	2	7.1
*Enterococcus thailandicus*	1	3.6
*Enterococcus villorum*	1	3.6
Total	28	100.0

**Table 3:** Proportions of ESBL-/AmpC-producing Gram-negative bacterial species identified by MALDI-TOF MS (*n* = 43).

ESBL-/AmpC bacteria	Frequency	Percentage (CI)
*Escherichia coli*	25	58.1
*Klebsiella pneumoniae*	12	27.9
*Klebsiella variicola*	2	4.7
*Citrobacter freundii*	2	4.7
*Aeromonas caviae*	1	2.3
*Morganella morganii*	1	2.3
Total	43	100.0

**Table 4:** AMR in study one bacterial isolates tested with 11 antimicrobials of clinical significance.

Antimicrobial	Class	Proportion of resistant bacteria, *N* (%)		
		Generic *E. coli* (*n* = 42)^a^	ESBL-/AmpC *E. coli* (*n* = 40)^d^	Enterococci (*n* = 42)
Trimethoprim sulphamethoxazole	Potentiated sulphonamides	16 (38.1)	29 (72.5)	^c^
Ceftriaxone	3^rd^ generation cephalosporins	4 (9.5)	31 (77.5)	^c^
Gentamicin	Aminoglycosides	0 (0.0)	28 (70.0)	4 (9.5)^b^
Ciprofloxacin	Newer quinolones	3 (7.1)	14 (35)	34 (81.0)
Imipenem	Carbapenems	0 (0.0)	3 (7.5)	^c^
Ampicillin	Penicillins	29 (69.0)	32 (80.0)	33 (78.6)
Tetracycline	Tetracyclines	20 (47.6)	26 (65.0)	37 (88.1)
Chloramphenicol	Phenicols	2 (4.8)	12 (30.0)	^c^
Linezolid	Oxazolidinones	^c^	^c^	39 (92.9)
Erythromycin	Macrolides	^c^	^c^	34 (81.0)
MDR⁣^∗^		16 (38.1)	40 (100.0)	41 (97.6)
MARI (average)		0.22	0.55	0.71

Abbreviation: MARI, multiple antibiotic resistance index.

⁣^∗^Bacterial multidrug resistance (MDR) defined as resistance to at least three different classes of antimicrobials.

^a^Commensal *E. coli* isolated from gut and cuticle on MacConkey agar without antibiotics.

^b^High-level gentamicin-resistant (HLGR) enterococci tested with 120 µg gentamicin disc.

^c^No testing done.

^d^40 ESBL-/AmpC-producing *E. coli* samples (25 from the guts and cuticle samples, and 15 from the 42 generic *E. coli*) were tested.

**Table 5:** AMR of enterococci (*n* = 66) and *E. coli* (*n* = 15) isolates from study two.

Antimicrobial	Class	Bacterial pathogen	Resistance, *n* (%)
Ciprofloxacin	Newer quinolones	*E. coli*	3 (20.0)
		Enterococci	61 (92.4)

Trimethoprim sulphamethoxazole	Potentiated sulphonamides	*E. coli*	13 (86.7)
		Enterococci	66 (100.0)

Tetracycline	Tetracyclines	*E. coli*	10 (66.7)
		Enterococci	64 (97.0)

Gentamicin	Aminoglycosides	*E. coli*	1 (6.7)
		Enterococci	66 (100.0)

Ampicillin	Penicillins	*E. coli*	13 (86.7)
		Enterococci	5 (7.6)

Meropenem	Carbapenems	*E. coli*	3 (20.0)
		Enterococci	24 (36.4)

**Table 6:** Genetic profile of AMR in *E. coli* and enterococci.

Study	Bacteria	Resistance gene(s)	Number of samples	Frequency (%)
Study one	Commensal *E. coli*	*TetA*	15	7 (46.7)
		*Tet (B, C, D, E, G)*		0 (0.0)
		*sul1*		4 (26.7)
		*sul2*		4 (26.7)
		*sul3*		3 (20.0)
	ESBL-/AmpC-*E. coli*	*TetA*	25	15 (60.0)
		*TetB*		8 (32.0)
		*Tet (C, D, E, G)*		0 (0.0)
		*sul1*		13 (52.0)
		*sul2*		8 (32.0)
		*sul3*		6 (24.0)
	Enterococci	*TetM*	40	33 (82.5)
		*TetL*		34 (85.0)

Study two	*E. coli*	*TetA*	15	2 (13.3)
		*TetB*		1 (6.7)
		*Tet (C, D, E, G)*		0 (0.0)
		*sul1*		4 (26.7)
		*sul2*		4 (26.7)
		*sul3*		0 (0.0)
	Enterococci	*TetM*	66	13 (19.7)
		*TetL*		0 (0.0)

## Data Availability

All manuscript data that supported the findings of this study are available from the corresponding author upon request.
